# IL-1β is a key cytokine that induces trypsin upregulation in the influenza virus–cytokine–trypsin cycle

**DOI:** 10.1007/s00705-016-3093-3

**Published:** 2016-10-06

**Authors:** I. L. Indalao, T. Sawabuchi, E. Takahashi, H. Kido

**Affiliations:** Division of Enzyme Chemistry, Institute for Enzyme Research, Tokushima University, Tokushima, 770-8503 Japan

## Abstract

Severe influenza is characterized by a cytokine storm, and the influenza virus–cytokine–trypsin cycle is one of the important mechanisms of viral multiplication and multiple organ failure. The aim of this study was to define the key cytokine(s) responsible for trypsin upregulation. Mice were infected with influenza virus strain A/Puerto Rico/8/34 (H1N1) or treated individually or with a combination of interleukin-1β, interleukin-6, and tumor necrosis factor α. The levels of these cytokines and trypsin in the lungs were monitored. The neutralizing effects of anti-IL-1β antibodies on cytokine and trypsin expression in human A549 cells and lung inflammation in the infected mice were examined. Infection induced interleukin-1β, interleukin-6, tumor necrosis factor α, and ectopic trypsin in mouse lungs in a dose- and time-dependent manner. Intraperitoneal administration of interleukin-1β combined with other cytokines tended to upregulate trypsin and cytokine expression in the lungs, but the combination without interleukin-1β did not induce trypsin. In contrast, incubation of A549 cells with interleukin-1β alone induced both cytokines and trypsin, and anti-interleukin-1β antibody treatment abrogated these effects. Administration of the antibody in the infected mice reduced lung inflammation area. These findings suggest that IL-1β plays a key role in trypsin upregulation and has a pathological role in multiple organ failure.

## Introduction

Multiple organ failure with vascular hyperpermeability is a common cause of death in severe seasonal and highly pathogenic influenza A virus infection. The condition is usually associated with hypercytokinemia and severe edema in the lung, heart, liver, kidneys and brain [1, 2]. In our previous studies, we proposed the hypothesis of an “influenza–cytokine–trypsin” cycle as one of the key mechanisms of vascular hyperpermeability and multiple organ failure in severe influenza [3–6]. In the process of influenza A virus entry into the cell, proteolytic conversion of the viral envelope fusion glycoprotein hemagglutinin (HA_0_) into HA1 and HA2 subunits by host cellular trypsin-type proteases is a pre-requisite for membrane fusion activity [5–10] because HA-processing protease is not encoded in the viral genome. Once viral infection ensues, ectopic pancreatic trypsin, which is one of the cellular HA-processing proteases and is expressed in limited amounts in various organs, such as the lungs, heart and brain, is markedly upregulated through the induction of proinflammatory cytokines [3–6, 9]. The upregulated trypsin potentiates viral multiplication in various organs, leading to cellular dysfunction, vascular hyperpermeability and fluid imbalance through proteinase-activated receptor-2 (PAR-2) [4, 11–13] and tissue damage [14, 15] with the involvement of matrix metalloproteases (MMPs) [10, 16, 17]. Trypsin is also reported to be crucial for infectivity of other viruses, such as rotaviruses, members of the family *Reoviridae*, and coronaviruses, members of the family *Coronaviridae* [18, 19], in addition to influenza A virus, a member of the family *Orthomyxoviridae*, and Sendai virus and Newcastle disease virus, members of the family *Paramyxoviridae*.

Pro-inflammatory cytokines, such as interleukin-1β (IL-1β), interleukin-6 (IL-6), and tumor necrosis factor α (TNF-α), are increased in the infected organs and blood, and such hypercytokinemia is considered a host defense response to influenza virus infection [20]. The presence of high levels of pro-inflammatory cytokines, however, impairs energy metabolism in mitochondria, leading to cellular dysfunction and organ failure [21]. Although we reported in our previous *in vivo* and *in vitro* studies [3, 4, 21] that pro-inflammatory cytokine(s) can trigger trypsin upregulation through the influenza–cytokine–trypsin cycle, it is still not clear whether there is a key cytokine that affects trypsin upregulation, followed by the cascades of multiple organ failure in severe influenza.

The effects of challenges with individual and multiple pro-inflammatory cytokines on the inflammatory responses in various organs have been reported not only in mice and rats [22–25] but also in various cell lines [26–31]. These studies highlighted the interactions between IL-1β, IL-6 and TNF-α, which contribute to disease progression. However, little or no information is available on cytokine cross-talk in trypsin upregulation.

The aim of the present study was to determine the presence of pro-inflammatory cytokine(s) cross-talk and its effects on trypsin upregulation, particularly in the lungs, which are the initial site of influenza virus replication. In addition to animal studies, we also used A549 cells, a human type II lung epithelial cell line, to confirm the results of the animal experiments. The A549 cell line is suitable for the experiments because these cells constitutively express IL-1 receptor and do not release natural IL-1β inhibitors [32].

## Materials and methods

### Animals and virus

Specific- pathogen-free 5-week-old C5B7BL/6J female mice were purchased from SLC (Saitama, Japan). The mice were treated according to the Guide for the Care and Use of Laboratory Animals (NIH Publication No. 85-23, 1996), and the study was approved by the Animal Care Committee of Tokushima University. Influenza virus A/Puerto Rico/8/34 (H1N1) (PR8) was kindly provided by The Research Foundation for Microbial Diseases of Osaka University (Kagawa, Japan). Under ketamine and xylazine anesthesia, 1 to 40 plaque-forming units (pfu) of PR8 in 15 µL of saline or saline alone as non-infection control were instilled intranasally in mice. In the cytokine administration experiments, mice were treated by intraperitoneal injection of 100 µL of a single cytokine or a combination of cytokines twice a day for three days. The cytokines used for treatment were mouse recombinant IL-1β, IL-6, and TNF-α (R&D Systems, Minneapolis, MN) at a dose of 40 μg/kg per day. The dose was within the range of cytokines found in the lungs after 25 pfu of PR8 infection and was also within the dose recommended in a previous study [22]. Mice were monitored daily for body weight and survival rate and were euthanized at 0, 3, 4, 6, and 8 days postinfection, and the lungs were extracted to measure the levels cytokines and trypsin.

To assess the pathological role of IL-1β in multiple organ failure, 100 µL of anti-mouse IL-1β goat polyclonal antibodies (R&D Systems) for neutralization, purified mouse IgG from normal mice (Wako Pure Chemical Industries, Ltd., Osaka, Japan) at 1 mg/mL, or saline as the vehicle was administered intraperitoneally to mice. After treatment for 1 hour, 25 pfu of PR8 in 15 µL of saline or saline alone was instilled intranasally. Mice were euthanized at day 7 postinfection, and lungs were processed for histopathological evaluation.

### Histological analysis

The lungs were perfused with 30-40 mL of saline through the right atrium of the heart, fixed with 10 % buffered formalin, pH 7.2, isolated, and embedded in paraffin. The paraffin block was cross-sectioned with 5 µm thickness using a microtome (Leica). Hematoxylin and eosin (HE) staining was done subsequently. Images were acquired with a microscope (BZ-X710; Keyence Corporation, Osaka, Japan). To analyze the image of a whole lung, partial lung images were combined using BZ-X analyzer software (Keyence), and the percentage of the whole lung composed of hypercellular areas associated with infiltration of inflammatory cells was determined using BZ-X analyzer software as described previously [33, 34].

### Cell culture

Human alveolar type II epithelial cell carcinoma (A549) cells were obtained from ATCC. The cells (3 × 10^5^/well) were cultured at 37 °C under 5 % CO_2_ in Dulbecco`s modified Eagle medium (DMEM) containing 10 % fetal bovine serum and 50 μg of gentamycin per ml. Cells were passaged every 2 days. After 24 hours of starvation, the cells were treated with 10 ng of human recombinant IL-1β per ml for 1 hour, washed three times with PBS(-), and then cultured in fresh serum-free medium for 8 or 24 hours. At the indicated time points, the cells and medium were collected.

To assess the role of IL-1β in the influenza–cytokine–trypsin cycle in A549 cells, human recombinant IL-1β at 10 ng/mL in serum-free medium was pre-treated with 600 ng of anti-human IL-1β monoclonal antibody (R&D Systems) per ml for neutralization, or with PBS(-) at 37 °C for 30 minutes. These reaction mixtures were added to A549 cells, and the cells were cultured for 1 hour for stimulation. The cells were then washed three times with PBS(-) and cultured in fresh serum-free medium for an additional 8 hours, and the mRNA levels of trypsin, IL-1β, IL-6, and TNF-α were measured.

### Enzyme-linked immunosorbent assay (ELISA)

Freshly isolated lungs were homogenized with 10 volumes of PBS(-) and centrifuged at 11,100×*g* for 20 minutes at 4 °C, and the supernatants were isolated. The lung extracts and media from A549 cell culture were subjected to ELISA analysis. The levels of IL-6, TNF-α and IL-1β, and trypsin were measured using ELISA kits (R&D Systems) according to the respective protocol provided by the manufacturer.

### Extraction of total RNA and real-time reverse transcription polymerase chain reaction (RT-PCR) analysis

Total RNA from A549 cells was isolated using an RNeasy Mini Kit (QIAGEN, Hilden, Germany) using the protocol supplied by the manufacturer. The cDNAs were synthesized using oligonucleotide primers and SuperScript III RT (Gibco BRL, Grand Island, NY). Real-time RT-PCR was used to measure expression of the human β-actin gene (accession number NM_001101.3), trypsin PRSS1 (accession number NM_002769.4), PRSS2 (accession number NM_002770.3), PRSS3 (accession number NM_002771.3), IL-1β (accession number NM_000576.2), IL-6 (accession number NM_000600.3), and TNF-α (accession number NM_000594.3).

The following primers were used in this study: β-actin, 5’-GCCGGGACCTGACTGACTACCTC-3’ (forward) and 5’-CTAGAAGCATTTGCGGTGGACGAT-3 (reverse); PRSS1, 5’-ATCCAGGTGAGACTGGGAGAGCACA-3’ (forward) and 5’-GTAGACCTTGGTGTAGACTCCAGGC-3’ (reverse); PRSS2, 5’-CCCCTTTGATGATGATGAC-3’ (forward) and 5’-AACTGTTCATTCCCCTCC-3’ (reverse); PRSS3, 5’-AGCGAACAGTGGGTGGTATC-3’ (forward) and 5’-GGCAGGTGAGGAGAGTTTGA -3’ (reverse); IL-1β, 5’-AAATACCTGTGGCCTTGGGC-3’ (forward) and 5’-TTTGGGATCTACACTCTCCAGCT-3’ (reverse); IL-6, 5’-GTAGCCGCCCCACACAGA-3’ (forward) and 5’-CATGTCTCCTTTCTCAGGGCTG-3’ (reverse); TNF-α, 5’-CCCAGGGACCTCTCTCTAATCA-3’ (forward) and 5’-GCTTGAGGTTTGCTACAACATG-3’ (reverse).

A SYBR Green PCR kit (Roche Diagnostics, Mannheim, Germany) and an ABI 7300 system (Applied Biosystems, Foster City, CA) were used to quantify these genes. The reaction conditions were set at 95 °C for 10 minutes, followed by 40 cycles of 30-second denaturation at 95 °C, 30-second annealing at 58 °C (for β-actin, PRSS1 and PRSS3), 55 °C (for PRSS2) or 60 °C (for IL-1β, IL-6, and TNF-α) and 50-second extension at 72 °C. The relative units were calculated from a standard curve and normalized to that of human β-actin.

### Statistical analysis

Data are expressed as mean ± standard deviation (SD) of at least three replicates, unless stated otherwise. Differences between groups were examined for statistical significance by Student’s *t*-test. A *P*-value less than 0.05 was considered statistically significant.

## Results

### Modes of upregulation of pro-inflammatory cytokines and ectopic trypsin in lungs by influenza A virus infection

**Fig. 1:**
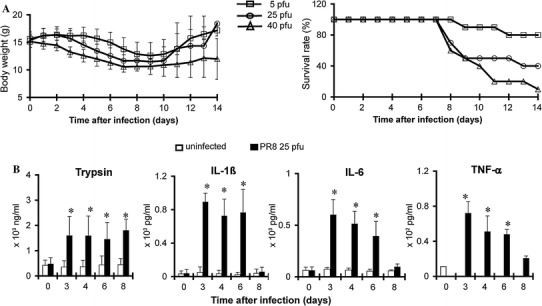
Effects of PR8 infection on body weight and survival rates (A) and the levels of pro-inflammatory cytokines and ectopic trypsin in the lungs (B). Anesthetized C5B7BL/6 J mice were infected intranasally with 5, 25, and 40 pfu of influenza virus PR8 or instilled saline as a control. Mice were sacrificed at days 0, 3, 4, 6, and 8 after infection. The levels of trypsin, IL-1β, IL-6, and TNF-α in the lungs were analyzed by ELISA. Data are the mean ± SD for 10 mice per group. *, P < 0.05 vs. the uninfected group (saline) by Student’s *t*-test

Mice were infected with influenza A virus PR8 to study the mode of upregulation pro-inflammatory cytokines and ectopic trypsin in the lungs in the early phase of infection. Based on the survival rates and changes in body weight at various doses of PR8 infection (Fig. 1A), we chose 25 pfu as the infection dose for subsequent experiments designed to measure the changes in cytokines and trypsin in the lungs.

IL-6 and TNF-α levels were increased significantly at day 3 postinfection and then gradually decreased afterwards. Meanwhile, the levels of IL-1β and trypsin were also increased significantly at day 3 postinfection, but the levels continued to be persistently high until day 8, although the high levels of IL-1β returned rapidly to the baseline (before infection) at day 8 (Fig. 1B). Interestingly, infected mice, particularly those infected with 25 and 40 pfu, showed signs of sickness (e.g., inactivity, ruffled fur, labored respiration, and body weight loss) after infection for 3 days, coinciding with the rise in pro-inflammatory cytokines and trypsin levels.

**Fig. 2:**
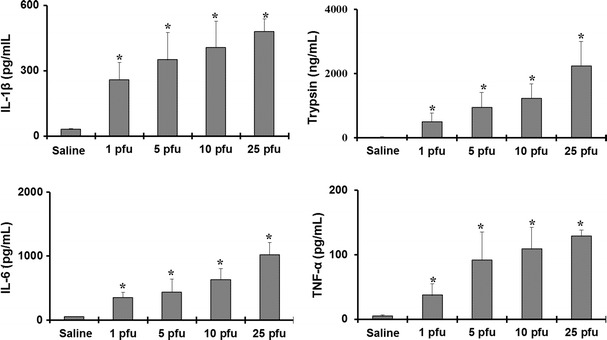
Dose-response profiling of PR8 virus on the upregulation of trypsin, IL-1β, IL-6, and TNF-α in the lungs. Each group (n = 3) received different doses of PR8. The levels of trypsin, IL-1β, IL-6, and TNF-α in the infected lungs were measured. Data are the mean ± SD. *, *P* < 0.05 vs. the uninfected group (saline) by Student’s *t*-test

Figure 2 shows the dose-response profile of the effect of PR8 virus infection on the upregulation of trypsin, IL-1β, IL-6, and TNF-α in the lungs at day 3 post-infection. The results indicate that upregulation of trypsin, IL-1β, IL-6, and TNF-α correlates closely with the viral dosage.

### Exogenously injected IL-1β alone or in combination with other cytokines induces similar morbidity characteristics and upregulates cytokines and trypsin in the lungs in the same manner as influenza A virus infection

**Fig. 3:**
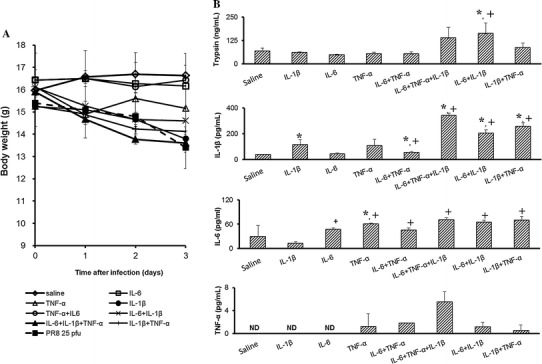
Effects of intraperitoneal injection of IL-1β and other cytokines on body weight and the levels of pro-inflammatory cytokines and trypsin in the lungs. IL-1β alone and IL-1β combined with other cytokines were injected intraperitoneally twice a day for three days, and the change in body weight was monitored (A). Data are the mean ± SD (n = 3), except data for PR8 (n = 10). The levels of pro-inflammatory cytokines and trypsin in the lungs at day 3 after infection with PR8 (25 pfu) were analyzed by ELISA (B). Data are the mean ± SD (n = 3). *, *P* < 0.05 vs. saline; ^**+**^, *P* < 0.05 vs. the IL-1β-injected group by Student’s *t*-test. ND, not detectable

To clarify the role of cytokines in the host cellular responses to infection, we analyzed the effect of intraperitoneal administration of IL-1β alone or IL-1β combined with IL-6 and/or TNF-α on the levels of ectopic trypsin and pro-inflammatory cytokines at day 3 post-treatment and measured changes in body weight. Animals treated with IL-1β alone or IL-1β combined with IL-6 and/or TNF-α became sick and lost weight in a manner similar to that observed in mice infected with 25 pfu of PR8. However, mice treated with IL-6 and/or TNF-α did not show any detectable symptoms or visible body weight loss (Fig. 3A). These findings suggest that IL-1β seems to play an important role in signal cascades in the pathogenesis of severe influenza virus infection.

Under the same experimental conditions, we further analyzed the levels of ectopic trypsin and pro-inflammatory cytokines in the lungs at day 3 post-treatment (Fig. 3B). Trypsin levels were not significantly increased following the administration of each cytokine but tended to increase following administration of IL-1β combined with IL-6 and/or TNF-α. In particular, the trypsin level induced by the combination of IL-1β plus IL-6 was significantly higher than that induced by IL-1β alone or saline. The combination of cytokines without IL-1β did not induce trypsin in the lungs. These results suggest that IL-1β is indispensable for trypsin upregulation and that IL-6 and/or TNF-α plays a co-stimulatory role in the effects of IL-1β.

IL-1β in the lungs increased significantly after administration of IL-1β or various cytokine combinations. Although IL-6 was not induced by IL-1β alone, it was induced significantly by IL-6, TNF-α and cytokine combinations including IL-1β.

### IL-1β-treated human alveolar A549 cells upregulate trypsin and increase pro-inflammatory cytokine secretion in a time-dependent manner

**Fig. 4:**
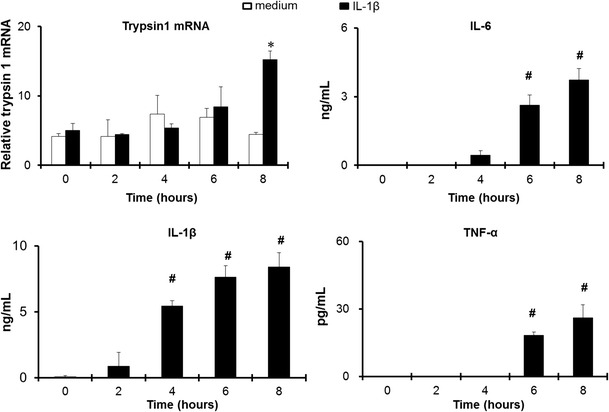
Kinetics of upregulation of trypsin, IL-1β, IL-6 and TNF-α in cultured A549 cells after treatment with human recombinant IL-1β. A549 cells were primed with 10 ng of recombinant human IL-1β per mL for 1 hour, washed with PBS(-), and then incubated in fresh serum-free medium containing IL-1β for 0 to 8 hours. Trypsin mRNA levels in the cell lysates and the levels of IL-1β, IL-6, and TNF-α in the culture medium were analyzed. Data are the mean ± SD (n = 3). *, *P* < 0.05 vs. the untreated group; #, *P* < 0.05 vs. 0 hour by Student’s *t*-test

**Fig. 5:**
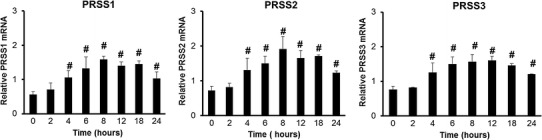
Gene upregulation of trypsin isoforms in A549 cells treated with human recombinant IL-1β. After 1 hour of incubation with 10 ng of IL-1β per mL at 37 °C, cells were collected to measure mRNA levels of human trypsin (PRSS) 1, 2, and 3 at various time points within 24 hours. #, *P* < 0.05 vs. 0 hour (before IL-1β addition) by Student’s *t*-test

To confirm the role of IL-β in trypsin upregulation in lung epithelial cells, the site of initial virus infection, human alveolar A549 cells were treated with 10 ng of IL-β per ml, and the expression levels of trypsin in the cells and the levels of secreted IL-1β, IL-6 and TNF-α in the medium were analyzed. The earliest response to the challenge was IL-1β secretion, detected at 2 hours in the medium, followed by secretion of IL-6 and TNF-α at 4 and 6 hours, respectively, with significant upregulation of trypsin 1 (PRSS1) mRNA, detected at 8 hours in the cells in the presence of IL-1β, IL-6 and TNF-α in the medium (Fig. 4). The levels of these cytokines and trypsin 1 increased continuously in a time-dependent manner during the 8-hour experiment. The other isoforms of human major trypsin mRNAs, such as PRSS2 and PRSS3, were also upregulated after IL-1β treatment in a manner similar to PRSS1, with peaks at 8 hours, and then gradually declined within 24 hours (Fig. 5). These findings indicate that IL-1β stimulates these trypsin isoforms gene expression equally in the lungs.

### Suppression of IL-1β by neutralizing monoclonal antibody markedly reduces trypsin, IL-1β, IL-6 and TNF-α upregulation

**Fig. 6:**
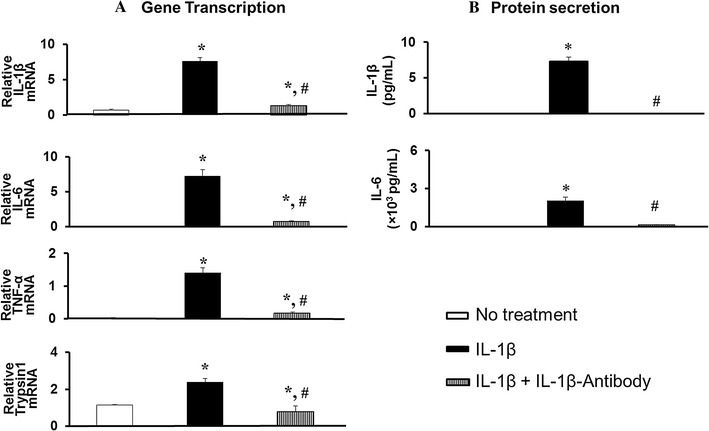
Suppressive effects of an anti-IL-1β-neutralizing monoclonal antibody on IL-1β-induced upregulation of trypsin and pro-inflammatory cytokines in A549 cells. IL-1β (10 ng/mL) in serum-free medium was reacted with or without anti-IL-1β monoclonal antibody (600 ng/mL) for 30 minutes at 37 °C. These reaction mixtures were added to A549 cells, and the cells were stimulated for 1 hour by incubation at 37 °C. The cells were then washed with PBS(-) and incubated for an additional 8 hours with fresh serum-free medium. After incubation, the mRNA levels of trypsin 1, IL-1β, IL-6, and TNF-α in the cells and the levels of IL-1β and IL-6 in medium were analyzed. *, *P* < 0.05 vs. the untreated group; #, *P* < 0.05 vs. the IL-1β-treated group by Student’s *t*-test

An IL-1β neutralization experiment was performed to add further support for the role of IL-1β in the upregulation of trypsin and pro-inflammatory cytokines in A549 cells (Fig. 6). IL-1β at 10 ng/mL was initially reacted with or without 600 ng of anti-human IL-1β monoclonal antibody per ml for neutralization in serum-free medium for 30 minutes. These reaction mixtures were then added to A549 cells, and the cells were stimulated for 1 hour, washed with PBS (-), and cultured for an additional 8 hours in serum-free medium. After incubation, trypsin, IL-1β, IL-6 and TNF-α mRNA levels in the cells and secreted IL-1β and IL-6 levels in the medium were analyzed. Anti-IL-1β monoclonal antibody effectively abrogated the upregulation of trypsin, IL-1β, IL-6, and TNF-α mRNAs in the cells and reduced the levels to those seen at baseline. Furthermore, IL-1β neutralization also prevented the rise in secreted IL-1β and IL-6 protein levels in the medium.

### Administration of anti-IL-1β neutralizing antibody significantly restrains development of inflammation in the lungs of mice infected with influenza A virus

**Fig. 7:**
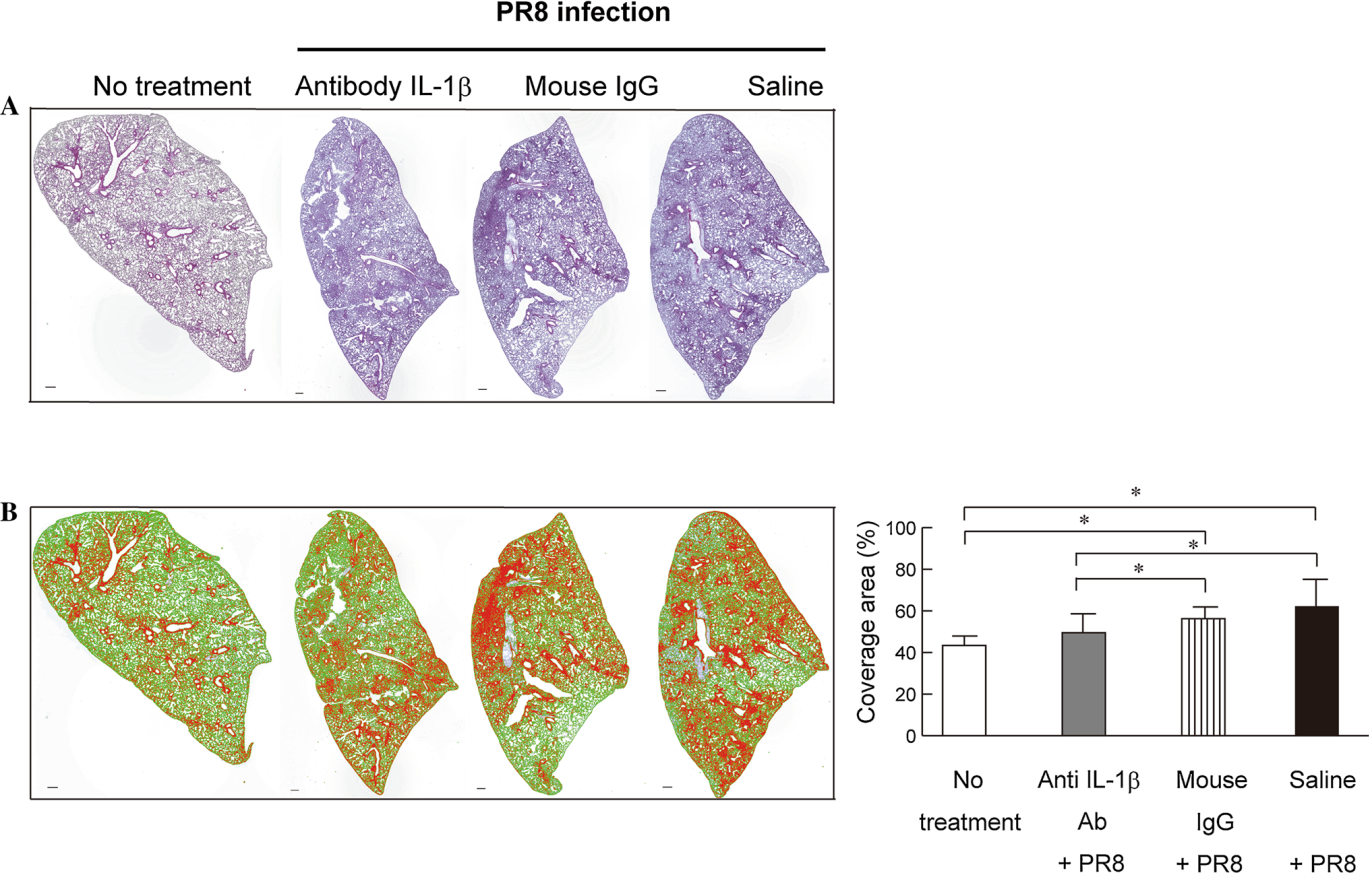
Administration of anti-IL-1β neutralizing polyclonal antibodies restrains development of inflammation in the lungs of mice infected with influenza A virus. Groups of mice received 100 μL of anti-mouse IL-1β neutralizing polyclonal antibodies, mouse IgG at 1 mg/mL, or saline prior to infection. After administration for 1 hour, 25 pfu of PR8 in 15 μL of saline or saline alone was instilled intranasally. At day 7 postinfection, mice were sacrificed and isolated lungs were prepared for hematoxylin and eosin staining. Images of histological analysis were acquired with a microscope (BZ-X710) with 2 × magnification (A). The hypercellular areas shown in red and the hypocellular areas shown in green were quantified in the lung. The percentage of the whole lung occupied by hypercellular areas was determined using BZ-X analyzer software (B). Scale bar, 300 μm. *, *P* < 0.05 by Student’s *t*-test

To verify the role of IL-1β in the influenza–cytokine–trypsin cycle and multiple organ failure in infected mice, anti-mouse IL-1β neutralizing polyclonal antibodies were administrated intraperitoneally before infection and pathohistological analysis in the lungs was conducted at day 7 postinfection, the time point of severe lung inflammation. In the lungs of infected mice treated with saline or mouse IgG as a negative control, inflammatory cells infiltrated and accumulated not only in bronchial regions and alveolar walls but also arterial and venous areas, resulting in abnormal thickening, resulting in strong staining with hematoxylin (Fig. 7A). Some lungs even exhibited damaged alveolar structure. In the lungs of infected mice treated with anti-IL-1β neutralizing polyclonal antibodies, however, the distribution and accumulation of inflammatory cells in the lungs were restricted. The hypercellular areas with inflammatory cell infiltration shown in red and the hypocellular areas shown in green were then quantified in the whole lung area (Fig. 7B). Histological quantification of the hypercellular area showed that anti-IL-1β neutralizing antibody treatment significantly suppressed the development of inflammatory cell aggregation when compared to the saline and control mouse IgG groups.

## Discussion

In the present study, we made several new observations: 1) among the pro-inflammatory cytokines IL-1β, IL-6 and TNF-α, which are induced in the early phase of PR8 infection, IL-1β plays a key role in trypsin upregulation in mouse lungs and human alveolar A549 cells. 2) IL-1β in combination with IL-6 and/or TNF-α effectively upregulated trypsin in mouse lungs, but the combination of cytokines without IL-1β did not. 3) IL-1β upregulated trypsin mRNAs (PRSS1, PRSS2, and PRSS3) equally in A549 cells in the presence of IL-1β, IL-6 and TNF-α. 4) Anti-IL-1β antibody neutralized IL-1β and efficiently suppressed the upregulation of pro-inflammatory cytokines and trypsin in A549 cells and also suppressed the development of inflammatory cell aggregation in the lungs of infected mice.

We reported previously that influenza A virus infection resulted in significant increases the levels of in IL-1β, IL-6, TNF-α, the viral hemagglutinin processing protease ectopic trypsin, and viral replication in the lungs of the host animal [3]. Upregulation of trypsin induces [Ca^2+^]_i_ mobilization via activation of PAR-2, followed by the loss of the intracellular tight junction protein zonula occludens-1, resulting in vascular hyperpermeability and multiple organ failure [3, 4, 6]. Furthermore, inhibitors of nuclear factor-kappa B (NF-κB) and activator protein 1 (AP-1) effectively suppress the upregulation of proinflammatory cytokines and ectopic trypsin and improve the survival rate of infected mice [3]. Based on these results, we proposed the influenza virus–cytokine–trypsin cycle hypothesis as one of the mechanisms of trypsin upregulation and vascular dysfunction in multiple organ failure with cytokine storm in severe influenza.

In this study, our interest was to identify major pro-inflammatory cytokines that can switch on trypsin upregulation in severe influenza A virus infection. We identified IL-1β as a key cytokine capable of inducing IL-1β, IL-6, TNF-α, and trypsin in mouse lungs and human alveolar A549 cells in the early phase of infection. We used four experiments to provide support for this result. First, we found that intraperitoneal injection of a mixture of pro-inflammatory cytokines (IL-1β, IL-6 and TNF-α) upregulated ectopic trypsin in the lungs, while a cytokine mixture that lacked IL-1β did not (Fig. 3B). Second, incubation of A549 cells in the presence of IL-1β stimulated IL-1β secretion initially, followed by secretion of IL-6 and TNF-α, and finally upregulated trypsin mRNAs in the presence of IL-1β, IL-6 and TNF-α in the culture medium (Fig. 4 and 5). Third, neutralization of IL-1β by monoclonal antibody against IL-1β almost completely suppressed such inductions *in vitro* (Fig. 6). Lastly, intraperitoneal administration of anti-IL-1β neutralizing polyclonal antibodies efficiently suppressed the development of inflammatory cell aggregation in the lungs of an infected mouse (Fig. 7).

The effects of cytokine synergism have been reported in various host cellular responses. Synergistic effects of IL-1β, IL-6 and TNF-α on inflammatory and stress mediators [22] and IL-1β and IL-17 on chronic lung inflammation after viral infection [23] have been reported. In particular, the interaction between IL-1β and IL-6 in many types of inflammation [24–26] and the mechanisms of synergism [27–29] have been studied extensively. Our results add support to these findings and confirm that IL-1β upregulates trypsin in the presence of IL-6 and/or TNF-α.

We reported previously that inhibitors of NF-κB and AP-1 effectively suppress the upregulation of proinflammatory cytokines, ectopic trypsin, viral replication, and endothelial dysfunction, resulting in a significant improvement in the survival of infected mice [3]. These transcription factors are activated by IL-1β signaling through mitogen-activated protein kinases (MAPKs) and the NF-κB pathway, as shown in previous studies [35–38]. These previous studies, including ours [3], support the present findings of the role of IL-1β in trypsin upregulation in combination with IL-6 and/or TNF-α, and also on the role of trypsin in the induction of various signal transductions in multiple organ failure. The pronounced suppressive effects of anti-IL-1β antibody on these effects also confirmed the crucial role of IL-1β.

**Fig. 8:**
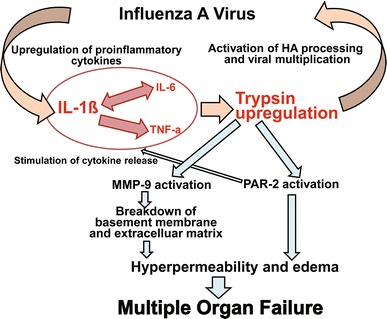
Diagram illustrating our hypothesis regarding the role of IL-1β in the influenza virus–cytokine–trypsin cycle and the pathogenesis of vascular hyperpermeability, tissue destruction and multiple organ failure in severe influenza. HA, hemagglutinin; PAR-2, proteinase-activated receptor-2; IL-1β, interleukin-1β; IL-6, interleukin-6; TNF-α, tumor necrosis factor-α; MMP, matrix metalloproteinase

The present study indicates a key role for IL-1β in trypsin upregulation in the early phase of influenza A virus infection in mouse lung. Figure 8 illustrates our hypothesis on the role of IL-1β in the influenza virus–cytokine–trypsin cycle and its synergistic interaction with IL-6 and/or TNF-α as indicated by arrow directions based on our results and the data reported [23–26]. Influenza A virus infection upregulates the proinflammatory cytokine IL-1β, followed by IL-6 and TNF-α, with synergistic interaction. Since upregulated trypsin efficiently converts pro-MMPs to active MMPs [16, 39], induction of trypsin and active MMPs synergistically degrades the vascular basement membrane and extracellular matrix, resulting in tissue damage, edema, and multiple organ failure. In addition, the upregulated trypsin in turn provokes cytokine release [40], cellular fluid imbalance, and loss of the intracellular tight junction protein zonula occludens-1 [3] via PAR-2 activation. These trypsin-based cascades result in vascular hyperpermeability and multiple organ failure in severe influenza.

Many studies have provided evidence for the therapeutic effects of controlling the balance of IL-1β within certain levels in various diseases by blocking IL-1β receptor or neutralization antibodies [23, 41–47]. Our *in vivo* experiment using anti-IL-1β neutralizing antibodies also demonstrated a similar outcome. In addition, our findings regarding the hypothesis illustrated in Figure 8 highlight the important effect of restraining IL-1β-stimulation to avoid trypsin-mediated disease aggravation. Moreover, further studies focusing on IL-1β directed synergic effects of pro-inflammatory cytokines on trypsin upregulation are needed to strengthen our proposed hypothesis. In addition, several hemagglutinin- processing proteases in human airway epithelium other than trypsin, such as TMPRSS2 and HAT, play roles in influenza virus multiplication [48]. We cannot deny the possibility that upregulation of IL-1β and other pro-inflammatory cytokines by infection also induces these processing proteases. Therefore, further studies on the effects of inflammatory cytokines, including IL-1β on these hemagglutinin processing proteases are needed to understand the pathogenesis of multiple organ failure in severe influenza.
