# Correction to: IL-1b is a key cytokine that induces trypsin upregulation in the influenza virus–cytokine–trypsin cycle

**DOI:** 10.1007/s00705-018-4043-z

**Published:** 2018-09-28

**Authors:** I. L. Indalao, T. Sawabuchi, E. Takahashi, H. Kido

**Affiliations:** Division of Enzyme Chemistry, Institute for Enzyme Research, Tokushima University, Tokushima, 770-8503 Japan

## Correction to: Arch Virol (2017) 162:201–211 10.1007/s00705-016-3093-3

The original version of this article unfortunately contained a mistake in the “Enzyme-linked immunosorbent assay (ELISA)” section of “Materials and methods” third paragraph. The corrected paragraph is updated here.

The levels of IL-6, TNF-a and IL-1b, and trypsin were measured using ELISA kits (R&D Systems **for the cytokines and MyBioSource for trypsin**) according to the respective protocol provided by the manufacturer.

